# Comparison between MR and CT imaging used to correct for skull-induced phase aberrations during transcranial focused ultrasound

**DOI:** 10.1038/s41598-022-17319-4

**Published:** 2022-08-04

**Authors:** Steven A. Leung, David Moore, Yekaterina Gilbo, John Snell, Taylor D. Webb, Craig H. Meyer, G. Wilson Miller, Pejman Ghanouni, Kim Butts Pauly

**Affiliations:** 1 Department of Bioengineering, Stanford University, Stanford, CA USA; 2 Focused Ultrasound Foundation, Charlottesville, VA USA; 3 Department of Biomedical Engineering, University of Virginia, Charlottesville, VA USA; 4 Department of Neurological Surgery, University of Virginia, Charlottesville, VA USA; 5 Department of Electrical Engineering, Stanford University, Stanford, CA USA; 6 Department of Radiology and Medical Imaging, University of Virginia, Charlottesville, VA USA; 7 Department of Radiology, Stanford University, Stanford, CA USA

**Keywords:** Biomedical engineering, Imaging techniques, Translational research

## Abstract

Transcranial focused ultrasound with the InSightec Exablate system uses thermal ablation for the treatment of movement and mood disorders and blood brain barrier disruption for tumor therapy. The system uses computed tomography (CT) images to calculate phase corrections that account for aberrations caused by the human skull. This work investigates whether magnetic resonance (MR) images can be used as an alternative to CT images to calculate phase corrections. Phase corrections were calculated using the gold standard hydrophone method and the standard of care InSightec ray tracing method. MR binary image mask, MR-simulated-CT (MRsimCT), and CT images of three ex vivo human skulls were supplied as inputs to the InSightec ray tracing method. The degassed ex vivo human skulls were sonicated with a 670 kHz hemispherical phased array transducer (InSightec Exablate 4000). 3D raster scans of the beam profiles were acquired using a hydrophone mounted on a 3-axis positioner system. Focal spots were evaluated using six metrics: pressure at the target, peak pressure, intensity at the target, peak intensity, positioning error, and focal spot volume. Targets at the geometric focus and 5 mm lateral to the geometric focus were investigated. There was no statistical difference between any of the metrics at either target using either MRsimCT or CT for phase aberration correction. As opposed to the MRsimCT, the use of CT images for aberration correction requires registration to the treatment day MR images; CT misregistration within a range of ± 2 degrees of rotation error along three dimensions was shown to reduce focal spot intensity by up to 9.4%. MRsimCT images used for phase aberration correction for the skull produce similar results as CT-based correction, while avoiding both CT to MR registration errors and unnecessary patient exposure to ionizing radiation.

## Introduction

Transcranial focused ultrasound (FUS) is an incisionless therapeutic modality that has human applications for thermal ablation^1–4^ as an alternative to open brain surgery, neuromodulation^5–7^ to probe the circuitry of the brain, and blood brain barrier opening^8–11^ to improve targeted drug delivery into the brain. In these applications, ultrasound is transmitted through the intact skull and focused deep into brain tissue to induce thermal or biomechanical effects. These effects can either be transient or irreversible depending on the parameters used during treatment.

Focusing ultrasound through the intact skull is challenging because the skull defocuses the ultrasound beam and shifts the focal spot away from the target. Correcting for the skull’s effects is highly complex due to the skull’s heterogeneity within and across patients, often varying in thickness, shape, size, and bone composition. Differences between skulls result in a broad range of transcranial ultrasound efficiencies across patients, such that the same applied power can lead to a four-fold range in temperature rise at the focal spot^12^.

Many methods have been proposed to account for the aberrating effects of the skull in order to refocus the focal spot. These methods include ray tracing^13–15^, finite difference time domain (FDTD)^14,16–26^, pseudo-spectral time domain (PSTD)^27–31^, and hybrid angular spectrum (HAS)^12,32–37^. Although these methods are different in theory and implementation, they have one similarity: they need to computationally model the skull to estimate and correct for its aberrating effects. For clinical ablation treatments, computed tomography (CT) images are used to create these computational skull models. However, the use of CT has several downsides. First, pre-operative CT images need to be registered to intra-operative magnetic resonance (MR) images, thus potentially leading to registration errors. Errors in registration could compromise phase corrections and lead to degradation of beamforming performance. Second, CT exposes patients to ionizing radiation, which is undesirable, for example, in pediatric populations. It is also undesirable for use with neuromodulation, as volunteers may be discouraged from participating in experiments needed to translate the technology for human use. Third, using CT images increases treatment workflow overhead, requiring separate imaging sessions to acquire all pre-operative images.

An alternative to CT is MR imaging, a safe, non-ionizing imaging modality. In recent years, developments in MR imaging of bone have been able to produce images with CT-like bone contrast^38–43^. These MR imaging techniques use ultrashort echo time (UTE), zero echo time (ZTE), or T1-weighted magnetization preparation rapid acquisition gradient echo (T1w MPRAGE) sequences to acquire bone signal before it decays rapidly due to T2* effects. T1w MPRAGE-derived skull models performed comparably to CT-derived skull models when simulating a single element transducer^43^, and UTE-derived skull models performed comparably to CT-derived skull models when simulating a hemispherical head transducer^41,42^. Furthermore, when using the standard of care InSightec ray tracing method for phase correction, UTE-derived binary images of the skull performed comparably with CT images^44^. In both Guo et al. and Miller et al., MR proton resonance shift thermometry (MR thermometry) was used to evaluate non-simulated experimental results. However, due to the method’s underlying resolution of 1.09 × 2.18 × 3 mm, it outputs data that averages the intensity of the focal spot and quantizes positioning errors into step sizes that are relatively large compared to a nominal focal spot size of 1.5 × 1.5 × 3 mm.

In this study, we directly compared the effectiveness of using CT versus MR-simulated-CT (MRsimCT) images for beamforming in an InSightec Exablate phased array transducer. We performed 3D hydrophone raster scans to measure the resulting focal spots and evaluated them using six metrics: pressure at the target, peak pressure, intensity at the target, peak intensity, positioning error, and focal spot volume. Additionally, we investigated the effects of CT misregistration on beamforming to emulate potential registration errors introduced during a clinical treatment. This provided a clearer comparison between the uses of CT and MRsimCT for treatment planning.

## Results

### MR images with different contrasts

**Figure 1:**
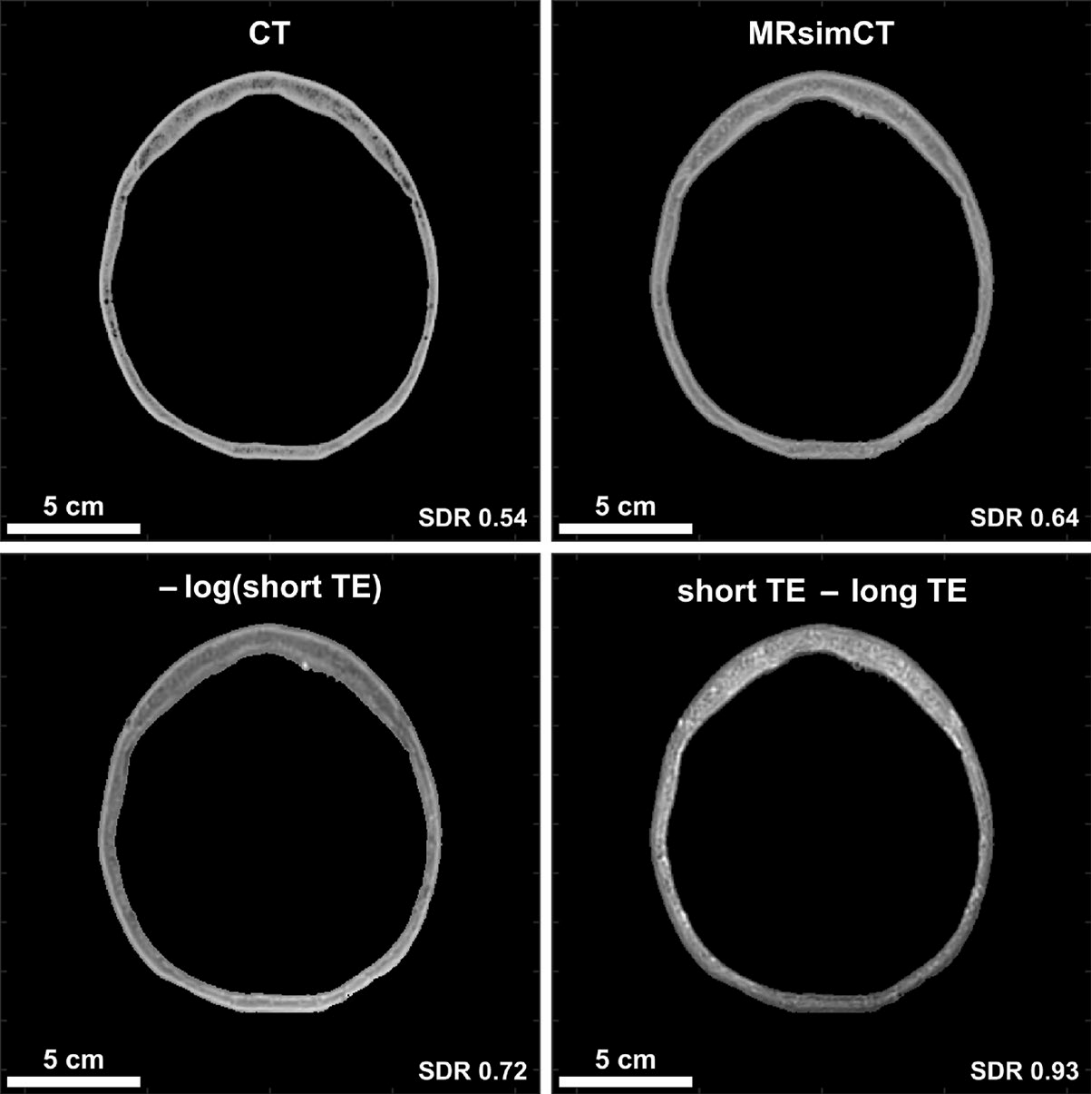
CT contrast of the skull compared to various MR contrasts (skull A). Cortical and trabecular bone contrast is clearly depicted by CT and may be preserved by two of the three MR post-processing methods. The units for MRsimCT, –log(short TE), and short TE–long TE are not the same, thus different windowing and leveling were used. Scale bars and skull density ratios (SDRs) are shown at the bottom of each image. MRsimCT was the preferred choice based on bone contrast, minimal SDR change compared to CT, minimal background signal bias, and generalizable post-processing. Therefore, it was used for the remainder of this study.

Three different image contrasts were generated using the acquired MR data: MRsimCT^38^, –log(short TE)^39^, and short TE–long TE^38^ (Fig. 1, Supplementary Figs. 1, 2). Qualitatively, all three contrasts preserved the overall skull shape, size, and thickness. Of the three options, MRsimCT achieved the most similar skull density ratio (SDR) as the CT, followed by –log(short TE), then short TE–long TE. SDR is a measure of skull homogeneity, with a value of 0 signifying low homogeneity and a value of 1 signifying high homogeneity. Changes in SDR compared to the CT suggest that bone composition information was lost.

Amongst the three MR contrasts of MRsimCT, –log(short TE), and short TE–long TE, MRsimCT was the preferred choice based on bone contrast, minimal SDR change compared to CT, and minimal background signal bias (Fig. 1, Supplementary Figs. 1–3). Background signal bias was due to RF transmit and receive nonuniformities, coil sensitivities, and/or coil shading. Although –log(short TE) also preserved some bone contrast, the remaining background bias would be a source of error when mapping from image values to acoustic properties. The residual background bias may be an artifact of the bias correction algorithm^39^ because it was observed in both ex vivo and in vivo images^39^. Furthermore, the bias correction algorithm required substantial parameter tuning to yield sufficient efficacy with the input images. Therefore, it may need to be tailored to each set of MR acquisition parameters. In contrast, the computation of short TE–long TE can be done rapidly, though it also exhibits residual background signal bias (Fig. 1, compare the anterior and posterior portions of the skull). Unlike the other two options, MRsimCT images exhibit minimal background signal bias likely because of its post-processing:

1 $$MRsimCT \approx \frac{{M}_{{TE}_{short}}-{M}_{{TE}_{long}}}{{M}_{{TE}_{short}}+{M}_{{TE}_{long}}}= \frac{{S}_{{TE}_{short}}B-{S}_{{TE}_{long}}B}{{S}_{{TE}_{short}}B+{S}_{{TE}_{long}}B}=\frac{{S}_{{TE}_{short}}-{S}_{{TE}_{long}}}{{S}_{{TE}_{short}}+{S}_{{TE}_{long}}},$$

where M_TEshort_ and M_TElong_ are the magnitude images from the short and long echo times, S_TEshort_ and S_TElong_ are the tissue signals from the short and long echo times, and B is the spatially nonuniform background bias due to RF transmit and receive nonuniformities, coil sensitivities, and/or coil shading. The magnitude images are a result of the underlying tissue signal weighted by the background bias term. From the MRsimCT post-processing, the bias term can be factored out from the numerator and denominator and removed from the overall expression.

**Figure 2:**
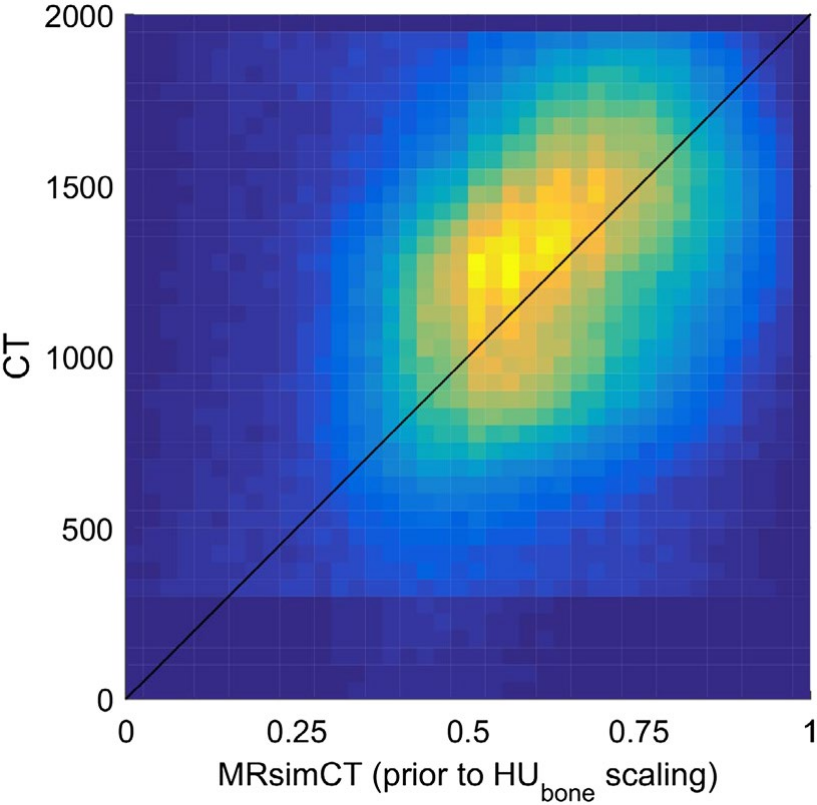
Relationship between CT and MRsimCT values. MRsimCT values prior to HU_bone_ scaling in (2) span a range from 0 to 1 and can be used as an estimate for bone fraction. For the CT parameters used in this study, pure bone was calculated to have a value of 2000 HU^36^. The black line depicts the nominal relationship between CT and MRsimCT values. Because only three ex vivo skulls were used in this study, using a linear regression to predict CT HU from MRsimCT values may overfit to the data. Therefore, the nominal linear relationship was used to predict CT HU instead.

MRsimCT was used for the remainder of this study for calculating phase corrections using the InSightec algorithm. Because only three skulls were used in this study, a nominal linear relationship between CT and MRsimCT values was used to estimate CT Hounsfield units (HU) (Fig. 2). Linear regressions fit to the data^39,41^ may also be used, though they are suitable only when large amounts of data are available. Otherwise, the linear regression may overfit to the data and result in loss of generalizability.

### 3D raster scans of phase corrected focal spots

**Figure 3:**
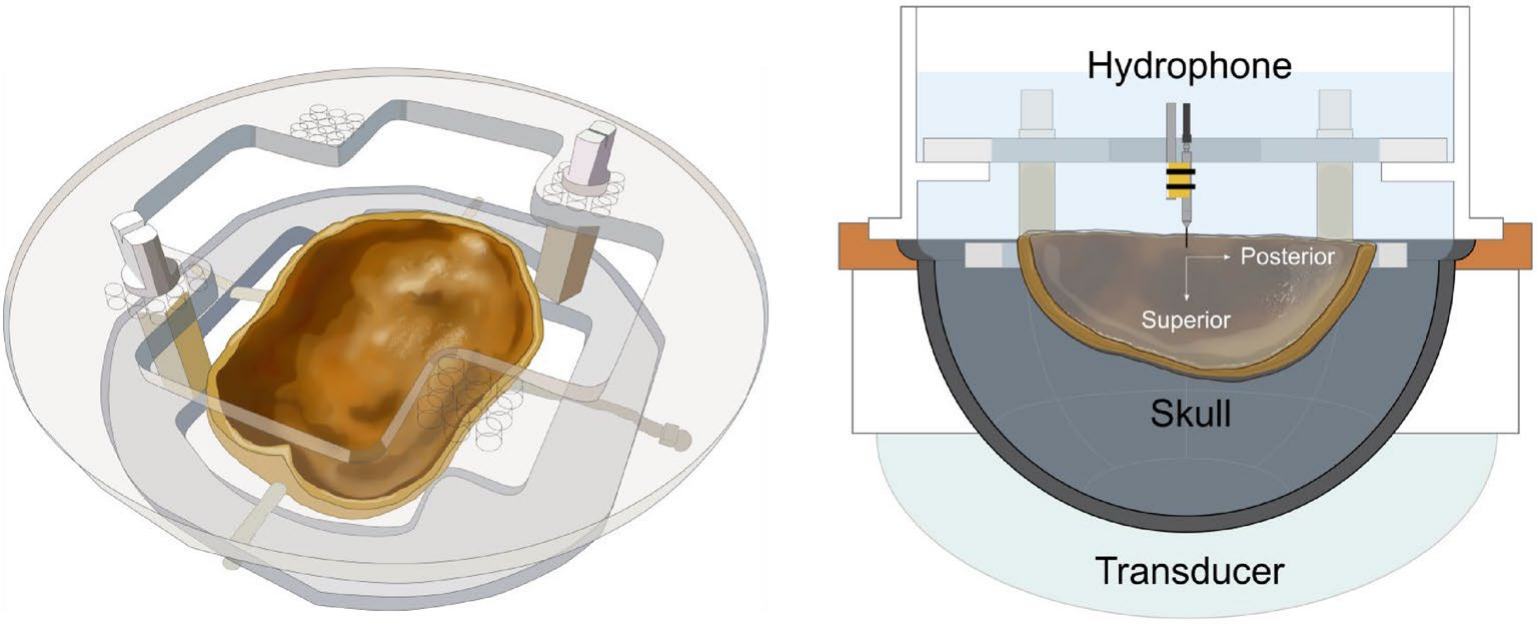
Experimental setup used in this study. Three ex vivo skulls were used for experimentation. Each of the three skulls was fixed to a head frame to ensure consistent positioning. The head frame was secured in an InSightec 670 kHz hemispherical phased array transducer. Data were acquired with a needle hydrophone and 3-axis positioner system. Illustrations were drawn by Sarah Hwang.

**Figure 4:**
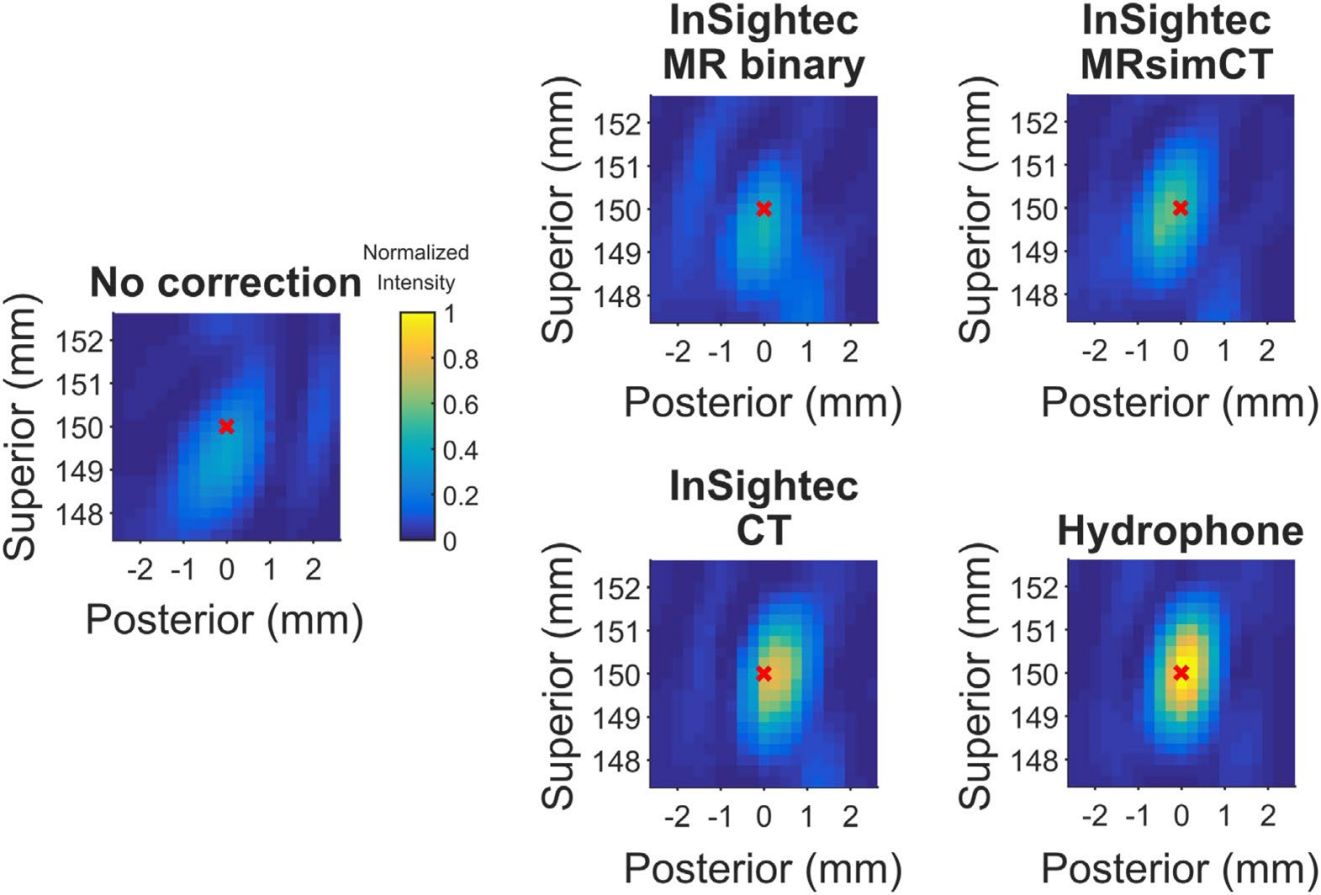
2D cross section raster scans of the refocused focal spot (skull A). The sagittal view is shown. These cross sections are a subset of the 3D volume scans acquired. A red x marks the position of the target, which was placed at the geometric focus. The focal spot maximum may be out of plane. Figure 5 shows quantitative metrics for all three skulls.

**Figure 5:**
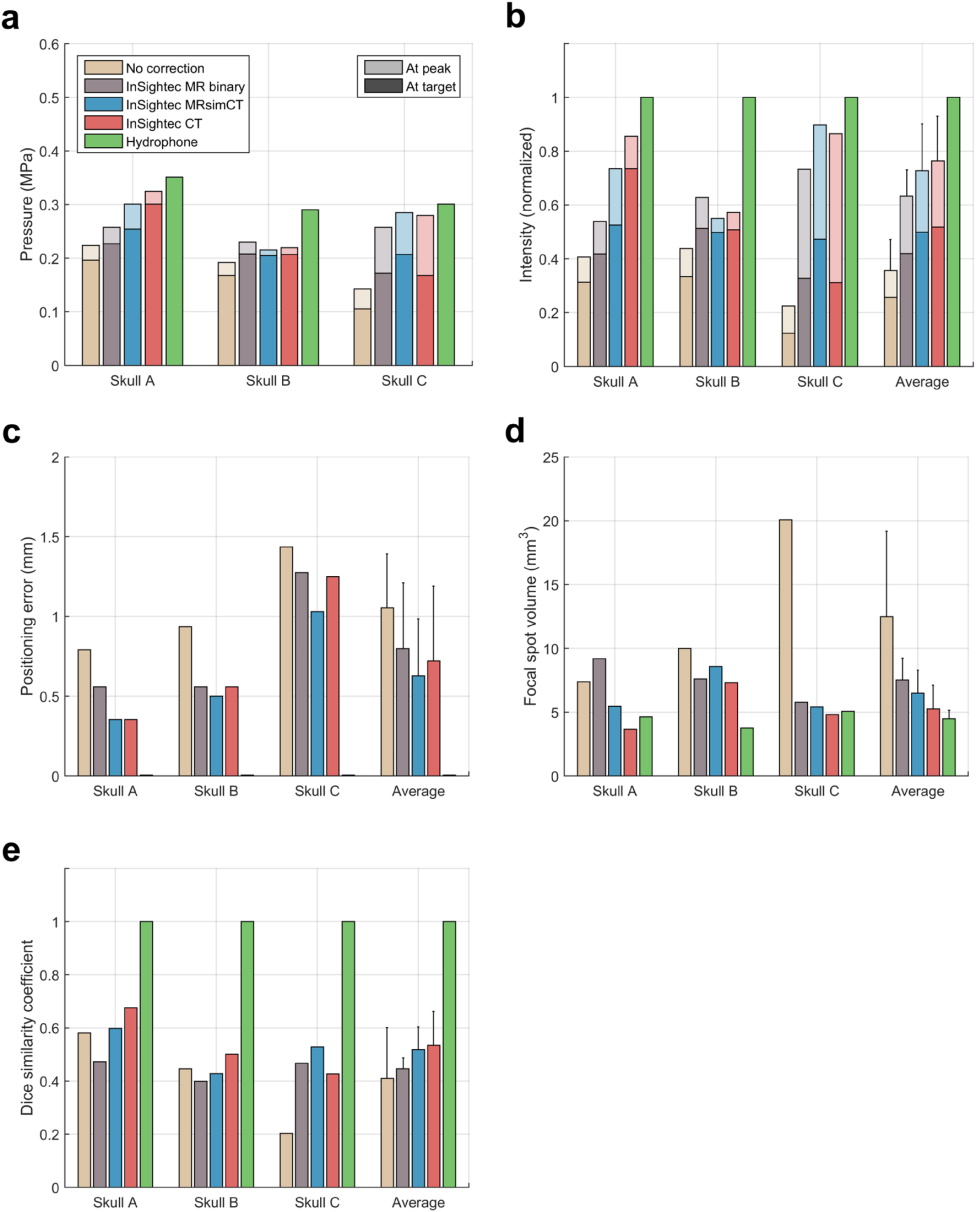
Beamforming performance of different image types with the target placed at the geometric focus. (**a**) Target and peak pressure (shown with darker and lighter colors, respectively) when operating the transducer at 20 W of electrical power. (**b**) Target and peak intensity normalized to the hydrophone method. (**c**) Focal spot positioning error. (**d**) Focal spot volume. (**e**) Dice similarity coefficient. Standard deviation bars are shown.

3D hydrophone raster scans were acquired for five sets of phase corrections: no correction, InSightec MR binary image mask, InSightec MRsimCT, InSightec CT, and hydrophone. The experimental setup is shown in Fig. 3, and example normalized intensity images from skull A are shown in Fig. 4. Three-plane cross sections of the focal spots can be found in Supplementary Figs. 4–6. With no phase correction, the focal spot was defocused and displaced from the geometric focus. The phase correction methods were able to recover the focal spot at or near the geometric focus, with varying pressures and positions. Using the hydrophone method for comparison, the presence of the skull reduced transmitted pressure to 25 ± 3% compared to free field. The focal spots were evaluated using six metrics, which are shown in Fig. 5 and summarized in Table 1.

**Table 1:** Summary of results for the phase corrected focal spots (target at the geometric focus).

	No correction	InSightec MR binary	InSightec MRsimCT	InSightec CT	Hydrophone
Target pressure	0.16 ± 0.05 MPa	0.20 ± 0.03 MPa	0.22 ± 0.03 MPa	0.23 ± 0.07 MPa	0.31 ± 0.03 MPa
Peak pressure	0.19 ± 0.04 MPa	0.25 ± 0.02 MPa	0.27 ± 0.05 MPa	0.27 ± 0.05 MPa	0.31 ± 0.03 MPa
Target pressure (normalized to hydrophone)	50 ± 13%	64 ± 7%	71 ± 2%	71 ± 15%	100 ± 0%
Peak pressure (normalized to hydrophone)	59 ± 10%	79 ± 6%	85 ± 10%	87 ± 10%	100 ± 0%
Target intensity (normalized to hydrophone)	26 ± 12%	42 ± 9%	50 ± 3%	52 ± 21%	100 ± 0%
Peak intensity (normalized to hydrophone)	36 ± 12%	63 ± 10%	73 ± 17%	76 ± 17%	100 ± 0%
Positioning error	1.05 ± 0.34 mm	0.80 ± 0.41 mm	0.63 ± 0.36 mm	0.72 ± 0.47 mm	0.00 ± 0.00 mm
Focal spot volume	12.48 ± 6.71 mm^3^	7.53 ± 1.70 mm^3^	6.48 ± 1.82 mm^3^	5.26 ± 1.86 mm^3^	4.48 ± 0.67 mm^3^

When targeting the geometric focus, the InSightec MR binary image mask, InSightec MRsimCT, and InSightec CT achieved comparable phase correction performance with respect to normalized target intensity, normalized peak intensity, positioning error, and focal spot volume (Fig. 5, Table 1). A one-way repeated measures ANOVA was performed, followed by a Tukey’s multiple comparisons test. No statistical difference was observed for any of the metrics (Supplementary Table 1). When targeting 5 mm to the left of the geometric focus, the InSightec MRsimCT and InSightec CT continued to perform similarly, whereas the InSightec MR binary image mask experienced degradation in performance. In particular, the target and peak intensities for InSightec MR binary image mask substantially decreased compared to the geometric focus case (Supplementary Table 2, Supplementary Fig. 8). A one-way repeated measures ANOVA and Tukey’s multiple comparisons test were performed, and there was again no statistical difference between image types for any of the metrics (Supplementary Table 3).

To investigate whether the nominal linear relationship between CT and MRsimCT introduced systematic beamforming errors, we additionally calculated phase corrections using a linear relationship fit to the data (Supplementary Fig. 9). Target pressures were computed using the post hoc analyses described in the methods. There was no statistical difference between the nominal linear relationship and the linear relationship fit to the data.

### Effects of CT misregistration

**Figure 6:**
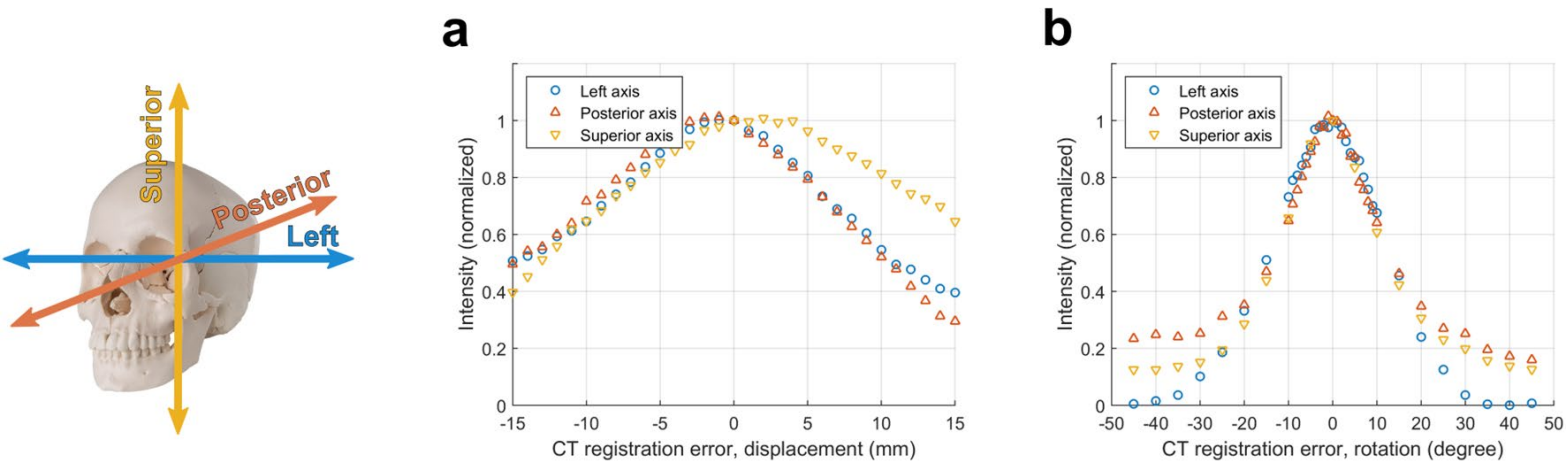
Effects of CT misregistration on normalized target intensity. (**a**) Displacement and (**b**) rotation errors were applied along one dimension only. Axes are colored according to their corresponding dimensions.

One benefit to using MR images is foregoing the need to perform image registration between CT and MR images. By registering pre-operative CT images with intra-operative MR images, misregistration errors can be introduced and reduce the effectiveness of estimated phase corrections. We performed error sensitivity analyses to estimate the influence of these registration errors. When errors were applied along one dimension, normalized target intensities decreased by approximately 10% within 4 mm of displacement (Fig. 6a) and within 4 degrees of rotation (Fig. 6b). 20% loss in normalized target intensity was observed within 6 mm of displacement and within 8 degrees of rotation. Phase correction performance was less sensitive to displacements along the superior axis, which may be because incidence angles were least affected by displacements along that axis. With rotation errors, performance was similarly sensitive along all three cardinal axes.

In practice, there exists an upper bound on the displacement and rotation errors due to misregistration. The upper bounds were approximated by performing manual registrations between pre-operative CT and intra-operative MR images, then evaluating the variability across multiple registrations. For two patients, manual registrations were performed five times each. Each registration was used as “ground truth” while the other four registrations were used to calculate the mean absolute registration errors. Across all patient registrations, the mean displacement errors were less than 0.4 mm and the mean rotation errors were less than 2 degrees. In keeping with the error analysis resolution of 1 mm and 1 degree for displacement and rotation, respectively, we assumed negligible displacement error and focused our analyses on combinations of rotation errors along three different axes. When rotation errors were applied in two dimensions, the maximum normalized target intensity loss within a ± 2 degree range was 8.8%. With rotation errors in three dimensions, the maximum normalized target intensity loss within a ± 2 degree range was 9.4%.

## Discussion

In this study, we compared the quality of several MR contrasts for bone imaging and directly compared the efficacy of several image types for correcting skull-induced phase aberrations. Although the MR binary image mask, MRsimCT, and CT images performed similarly when targeting the geometric focus, MR binary image mask performance substantially degraded when targeting 5 mm left of the geometric focus. MRsimCT shows great potential to be used as an alternative to CT. Qualitatively, the images exhibit CT-like bone contrast and preserve information on skull shape, size, thickness, and bone composition. Quantitatively, both sets of images resulted in similar target and peak intensities, positioning error, and focal spot volume. Investigation of CT misregistration showed that within the practical limits of error, the reduction in target intensity may be clinically relevant for ablation treatments.

### 3D raster scans of phase corrected focal spots

The beamforming performances of InSightec MRsimCT and InSightec CT were comparable based on six quantitative metrics. The same standard of care InSightec phase correction algorithm was used on both types of images, and the results suggest that MRsimCT images may already be suitable to be integrated into the existing clinical workflow for treatment planning.

Miller et al. previously reported that an MR binary image mask performed as well as CT when used with the InSightec ray tracing method^44^. Even with 10 mm of steering away from the geometric focus, the measured MR thermometry peak temperature rise and focal spot position were highly comparable, with less than 2.3% and 5.8% difference, respectively. The results from the present study show larger differences between MR binary image mask and CT, with 22.4% difference in peak intensity and 49.6% difference in positioning error when steering 5 mm left of the geometric focus. The increase in position error difference observed in this study could be due to the higher spatial resolution of hydrophone raster scans (0.25 mm isotropic) compared to MR thermometry (typically 1.09 × 2.18 × 3 mm). The increase in peak intensity difference remains unclear, though it could be due to the use of different ex vivo skulls or InSightec software versions. Unfortunately, we were unable to determine whether the same skulls or software versions were used in both studies.

Post hoc analyses showed that there was no statistical difference in beamforming performance between the nominal linear relationship and the linear relationship fit to the data, however, there are a couple of caveats to using the latter. One caveat is the potential loss of generalizability when there is a small amount of available data for model training and testing. Another caveat is the linear regression’s dependence on the image acquisition parameters. Both CT and MR acquisition parameters need to be kept constant in order to maintain a stable relationship between their image values. As an example, a change in MR receive gain during acquisition could drastically affect the mapping from MR to CT values. Changes in parameters such as resolution, MR flip angle, or CT tube voltage^45^ would also have non-negligible effects. Challenges exist in maintaining a fixed set of image acquisition parameters across operators and across imaging sites; despite a CT imaging protocol established by InSightec, there still exists variability in CT acquisition parameters. Similarly, there will also be challenges in maintaining a fixed set of MR acquisition parameters across operators and across sites. Thus, for future studies, accounting for image acquisition parameters for both MR and CT will be of great importance.

### Effects of CT misregistration

Up until this point, MRsimCT and CT were compared based on their best-case performances. In this study, both sets of images were well registered to the hemispherical transducer due to the use of fiducial markers in each head holder. In contrast, during clinical treatments, pre-operative CT images need to be registered to intra-operative MR images without the use of fiducial markers. Furthermore, registration to the intra-operative MR images is additionally challenging because of B_1_ inhomogeneity artifacts created by the ultrasound hemispherical transducer and water surrounding the head. This process can introduce registration errors, which may reduce the effectiveness of estimated phase corrections. Therefore, applying displacement and rotation errors to the registered CT images (and not introducing any registration errors to the MRsimCT images) allowed for a fairer comparison between MRsimCT and CT.

Registration errors reduced the normalized target intensity by a non-negligible amount that may be clinically relevant for ablation treatments. Although there exists a large parameter space of misregistration errors that lead to large losses in intensity, in practice, the errors are likely bounded to within ± 2 degrees of rotation along each dimension. Within this range, the maximum normalized target intensity loss was 8.8% when errors were present in two dimensions and 9.4% when errors were in three dimensions. For ablation treatments, this loss in intensity may not drastically affect patients whose skulls permit efficient transmission of ultrasound. However, for patients with highly aberrating or attenuating skulls, the intensity loss could be the difference between successfully or unsuccessfully achieving the desired ablative temperature at the target. The limitation is often not transducer output power, but rather, increased patient discomfort when operating at high powers.

### Study limitations

Although the MRsimCT versus CT results are encouraging, they should be considered with the limitations of this study. First, experiments were performed on only three skulls, a small sample size that may not be representative of the entire patient population. Investigation into additional skulls will need to be performed, ideally spanning a larger range of SDR values. Second, only two targets were used for phase correction. Although this may be sufficient for treatments requiring a small window of treatment, further investigation with electronic steering will be needed. Third, comparisons between the three types of images were performed using only the standard of care InSightec ray tracing algorithm. If a more accurate simulation method were used instead, there may be more pronounced differences in performance between the types of images. Although the conclusions presented in this study are applicable to the current standard of care, they may have to be revisited when considering new phase correction algorithms used in the future. These study limitations motivate important future work to acquire additional data and to characterize the effectiveness of MRsimCT versus CT for transcranial treatment planning.

## Conclusion

Compared to the use of CT images for treatment planning, the use of MRsimCT images produced similar results. Using MR images as an alternative to CT images has great relevance for transcranial FUS ablation treatments. It would reduce beamforming errors due to misregistration and avoid patient exposure to ionizing radiation. These improvements are also highly relevant for applications in neuromodulation and blood brain barrier opening, which are currently being translated for human use.

## Methods

### Skull imaging

Three ex vivo human skulls were used in this study. The protocol was approved by the Virginia State Anatomical Program and all research was conducted in accordance with the guidelines specified by the Virginia State Anatomical Program, the University of Virginia medical research protocols, and the University of Virginia Institutional Review Board for Health Sciences Research. Informed consent to use the donors’ skulls for scientific research were obtained from the donors and their next of kin.

The skulls were each fixed to a head frame prior to experimentation to ensure reproducible positioning between experiments (Fig. 3). Prior to imaging or other experimentation, the skulls were degassed overnight with an Abbess Instruments acrylic vacuum chamber. After degassing, the skulls were transferred to low density polyethylene bags in preparation for computed tomography (CT) and magnetic resonance (MR) imaging. The transfers were performed without exposing the skulls to air. A margin of 40 mm of degassed water between the skull and the bag material was used to avoid MR susceptibility artifacts.

Computed tomography (CT) scans for all three skulls were acquired using CT parameters approved by the InSightec patient screening imaging protocol (Table 2). The skull density ratios (SDR) are also reported. In general, SDR is a measure of bone composition homogeneity on a scale of 0 to 1, with higher SDR associated with higher homogeneity.

**Table 2:** CT parameters used for imaging the three ex vivo skulls.

Skull	Vendor	Tube voltage (kVp)	Reconstruction kernel	Compensation filter	Resolution	Skull density ratio
A	GE	120	BONEPLUS	Medium	0.5664 × 0.5664 × 0.625 mm	0.54
B	GE	120	BONEPLUS	Medium	0.5605 × 0.5605 × 0.625 mm	0.53
C	GE	120	BONEPLUS	Medium	0.5469 × 0.5469 × 0.625 mm	0.74

Ultrashort echo time (UTE) magnetic resonance (MR) images were acquired on a Siemens 3T Prisma MR scanner with a clinical 3T transmit-receive circularly polarized head coil (Siemens Healthcare GmbH). A 3D stack-of-spirals sequence^46^ was used with TE 50 μs and 2510 μs, TR 11 ms, flip angle 20 degrees, 1049 spiral interleaves, and matrix size 416 × 416 × 192 for a nominal resolution of 0.82 × 0.82 × 0.80 mm assuming no T2* decay. The UTE images were used to generate the MR-simulated-CT (MRsimCT) images^38^ using the following expression:

2 $$MRsimCT=\frac{{M}_{{TE}_{short}}-{M}_{{TE}_{long}}}{{M}_{{TE}_{short}}+{M}_{{TE}_{long}}}\times {HU}_{bone},$$

where M_TEshort_ is the magnitude image from the short echo time, M_TElong_ is the magnitude image from the long echo time, and HU_bone_ is the maximum HU of bone. HU_bone_ was calculated to be 2000 HU for the CT parameters used in this study using ρ_bone_ and mass attenuation coefficients for bone and water reported by the National Institute of Standards and Technology^36,47^ (Supplementary Fig. 10). We assumed an effective tube voltage of 60 kV for a 120 kVp spectrum because the spectrum data was not available to us. Prior to scaling by HU_bone_, the MRsimCT values spanned a range from 0 to 1 and can be used as an estimate for bone fraction. A value of 0 denotes water or a small proportion of bone in the voxel and a value of 1 denotes a large proportion of bone. Therefore, performing the HU_bone_ scaling gives a nominal relationship with which to predict CT HU. Although a linear regression between CT HU and MRsimCT values could also have been used, it may overfit to the data because only three skulls were used in this study. MR binary image masks were generated using a threshold of 0 HU. Bone voxels were set to 1000 HU and all other voxels were set to − 1000 HU^44^. Creating the MR binary image masks as described by Miller et al.^44^ presented the opportunity to benchmark the results from this study against the results from that study. This was of interest because both studies were performed at the University of Virginia using the same InSightec equipment. As an alternative to the MR binary image mask, additional complexity may be added; a multi-layered skull approach would be an intermediate step between the MR binary image mask method and the MRsimCT method. As seen in Fig. 1, there is enough bone contrast to delineate between cortical and trabecular bone. Either nominal HU values or average HU values calculated from a cohort of patients could be assigned to the bone layers. In this study, to use results from Miller et al.^44^ as points of comparison, an MR binary image mask was used instead of a multi-layered image mask.

Two other MR contrasts were investigated in this study: –log(short TE)^39^ and short TE–long TE^38^. Prior to calculating –log(short TE), the spatially nonuniform background bias was removed from the magnitude images using a bias correction algorithm reported by Wiesinger et al.^39^ (for these data, K = 60 levels of resolution and a low pass filter three times larger than the smallest region of interest were used). For the short TE–long TE computation, background bias was not removed.

### Experimental setup

The skull and head frame were secured in a 670 kHz InSightec Exablate 4000 hemispherical phased array transducer and positioned in the clinical orientation; the anterior portion of the skull was closest to the anterior portion of the transducer. An Onda HNA-0400 needle hydrophone was mounted on an Onda 3-axis positioner system and used to perform 3D raster scans of the beam profile.

Prior to skull sonication, the position of the geometric focus in water was determined using a series of 2D raster scans. All transducer elements were turned on, and the focal spot position was localized by alternating between axial, coronal, and sagittal scan planes and centering the system on the position with maximum pressure. A three-plane cross section of the focal spot in water is shown in Supplementary Fig. 11.

The InSightec workstation was operated in research mode and was supplied phase corrections prior to each sonication. All elements were fired simultaneously while the hydrophone was rastered in a 5 × 5 × 5 mm field of view grid. This 3D grid was acquired with 21 individual 5 × 5 mm sagittal scans with in-plane and through-plane step sizes of 0.25 mm. For each skull, data for all 21 sagittal scans and for all phase correction schemes were acquired on the same day. The pulsing scheme was 2 ms on, 50 ms off using 20 W of electrical power. An Agilent DS07012B oscilloscope and the Soniq software from Onda were used to record the peak to peak voltages, which were then converted into pressure and intensity amplitudes.

### Skull registration

To accurately model the experimental setup in the InSightec system, the position and orientation of the skull relative to the transducer was determined. Registration between the skull and head frame was not necessary, because their relative positioning and orientation were captured in the CT and MR images. The head frame was fabricated according to computer aided design (CAD) specifications, in which the position of the head frame relative to the transducer was also specified. Eight 2 mm diameter tantalum beads were epoxied onto the head frame at specified locations denoted in the CAD. These beads were installed prior to imaging and served as fiducial markers to register the images to the transducer (Supplementary Fig. 12). The centroid of each bead was used as its position in image space. A singular value decomposition-based least squares registration^15,37,48^ was performed to achieve point-wise registration between fiducial marker positions in image and transducer space. The transformation matrix that registers the two coordinate systems is:

3 $${P}_{transducer}=R{P}_{image}+Tr,$$

where P_transducer_ is a 3 × 8 matrix of fiducial marker positions in transducer space, P_image_ is a 3 × 8 matrix of fiducial marker positions in image space, R is a 3 × 3 rotation matrix, and Tr is a 3 × 8 translation matrix. The rotation matrix R is calculated as

4 $$R=U{V}^{T},$$

where U and V are the unitary matrices from the singular value decomposition of the matrix H

5 $$SVD\left(H\right)=U\Lambda {V}^{T},$$

6 $$H=\left({P}_{image}-\frac{1}{8}\sum_{i=1}^{8}{p}_{image,i}\right){\left({P}_{transducer}-\frac{1}{8}\sum_{i=1}^{8}{p}_{transducer,i}\right)}^{T},$$

and where p_image,i_ is a 3 × 1 position vector of the ith fiducial marker in image space, p_transducer,i_ is a 3 × 1 position vector of the ith fiducial marker in transducer space, and T is the transpose operator. With values for P_image_, P_tranducer_, and R, the translation matrix Tr can then be calculated using (3). The resulting transformation matrix was used to resample the images into transducer space to create a set of registered images. Axial slices with 0.5 × 0.5 × 1 mm resolution were used for the MR binary image mask, MRsimCT, and CT images.

### Phase correction methods

The geometric focus and 5 mm left of the geometric focus were selected as targets, and four different methods were used to estimate phase corrections. These methods were (1) InSightec ray tracing with MR binary image mask inputs, (2) InSightec ray tracing with MRsimCT image inputs, (3) InSightec ray tracing with CT image inputs, and (4) hydrophone.

The InSightec ray tracing method requires computational models of the skulls to compute phase corrections. These skull models were generated using either MR binary image mask, MRsimCT, or CT images of the skulls, which were first registered to the transducer as described above.

#### InSightec ray tracing method

Although the InSightec ray tracing method remains proprietary, we were able to calculate phase corrections by using the InSightec workstation in clinical mode. After uploading the registered MR binary image mask, MRsimCT, or CT images, we followed the clinical workflow for treatment planning (Supplementary Fig. 13). Because the skull was already registered to the transducer in these images, no further registration was necessary. Under the hood, the InSightec workstation maps image voxel values to acoustic properties, which are then used to calculate phase corrections. The phase corrections could be found in the system logs.

#### Hydrophone method

While the InSightec method requires models of the skull to compute phase corrections, the hydrophone method directly accounts for skull aberrations using experimental data. Individual elements were fired sequentially, and their time series signals were measured with the hydrophone positioned at the geometric focus. The pulse duration used was 3 ms at 375 W electrical power, the sampling frequency was 15 MHz, and the sampling duration was 7 ms. Data were acquired for all 1024 transducer elements using a TiePie Handyscope HS6 Diff and a Panametrics model 5676 preamplifier.

### Quantitative metrics

Six quantitative metrics were used to evaluate phase correction performance. Focal spot pressures were indexed at the target position and at the position of peak pressure. Intensities were calculated and normalized to the results from the hydrophone phase correction method, the gold standard for recovering maximum intensity. This normalization removed the influence of skull-dependent attenuation and allowed phase correction performance to be compared across skulls. Positioning errors were calculated as the difference between the target position and the focal spot position, defined to be at the voxel of peak pressure. Focal spot volume was measured at pressure full width half maximum.

### Post hoc analyses

Post hoc analyses of the (i) MRsimCT to CT relationship and (ii) CT misregistration were performed. To perform these analyses, phase corrections were applied to single element time series data previously acquired with the hydrophone method, detailed in the “Phase correction methods” section. By the superposition principle, the net focal spot pressure is equivalent to the sum of pressures contributed by the individual elements. Because the single element data were only acquired at the target position, only the target pressure could be computed for these analyses. The target pressure resulting from any set of phase corrections can be determined by the following expression:

7 $$P= \sum_{i=1}^{1024}{s}_{i}(t){ e}^{j{\phi }_{i}},$$

where P is the pressure averaged over 100 cycles, s_i_ is the time series data for element i, and ${\phi}$_i_ is the phase correction for element i calculated with one of the methods. Analyses by this method removed the need to reacquire raster scan data for each set of new phase corrections and substantially reduced the number of data acquisitions needed.

### CT misregistration

To investigate the effects of CT misregistration, displacements and rotations were applied to the registered CT images prior to computing phase corrections. Misregistration errors can cover a large parameter space due to six degrees of freedom: three directions of displacement and three axes of rotation. Therefore, to reduce the complexity of analyses and data visualization, we first evaluated phase correction performance with errors applied in one dimension. Displacement errors were applied in 1 mm increments along the left–right, posterior-anterior, or superior-inferior axes. Rotation errors were applied along the same axes, with the origin centered at the focal spot target. Increments of 1 degree were applied up until ± 10 degrees, after which increments of 5 degrees were applied. Phase corrections were recalculated for each displaced or rotated skull model. Post hoc analyses were then performed to evaluate the effects of misregistration on the resulting target pressure.

## Supplementary Information

Supplementary Information.
