# Supplementary material for: Comparison between MR and CT imaging used to correct for skull-induced phase aberrations during transcranial focused ultrasound
Source: Sci Rep. 2022 Aug 4;12:13407. doi: 10.1038/s41598-022-17319-4 (PMC9352781; doi:10.1038/s41598-022-17319-4)
Supplement: Supplementary file 1 — Supplementary Information. [file 41598_2022_17319_MOESM1_ESM.docx]

Supplementary information to:

Comparison between MR and CT imaging used to correct for skull-induced phase aberrations during transcranial focused ultrasound

Steven A. Leung^1,*^, David Moore^2^, Yekaterina Gilbo^3^, John Snell^2,4^, Taylor D. Webb^5^, Craig H. Meyer^3,6^, G. Wilson Miller^3,6^, Pejman Ghanouni^7^, Kim Butts Pauly^1,5,7^

^1^ Department of Bioengineering, Stanford University, Stanford, California, USA

^2^ Focused Ultrasound Foundation

^3^ Department of Biomedical Engineering, University of Virginia, Charlottesville, Virginia, USA

^4^ Department of Neurological Surgery, University of Virginia, Charlottesville, Virginia, USA

^5^ Department of Electrical Engineering, Stanford University, Stanford, California, USA

^6^ Department of Radiology and Medical Imaging, University of Virginia, Charlottesville, Virginia, USA

^7^ Department of Radiology, Stanford University, Stanford, California, USA

* Corresponding author: stevenleung@stanford.edu

**Skull B**


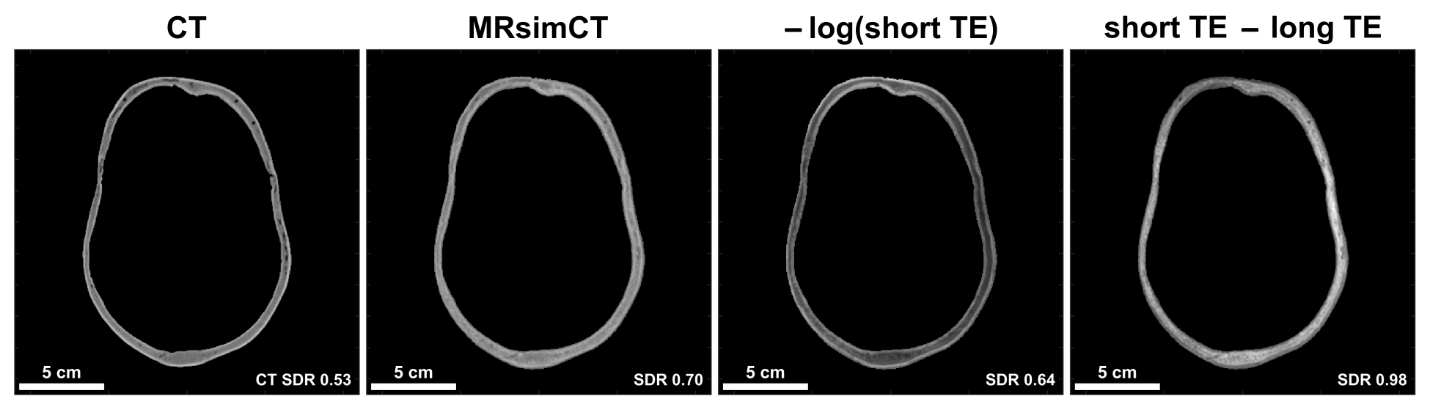


**Supplementary Figure 1.** CT contrast of the skull compared to various MR contrasts (skull B). Cortical and trabecular bone contrast is clearly depicted by CT and is preserved by two of the three MR post-processing methods. The units for MRsimCT, – log(short TE), and short TE – long TE are not the same, thus different windowing and leveling were used. Scale bars and skull density ratios (SDRs) are shown at the bottom of each image. Corresponds with Figure 1 in the main text.

**Skull C**


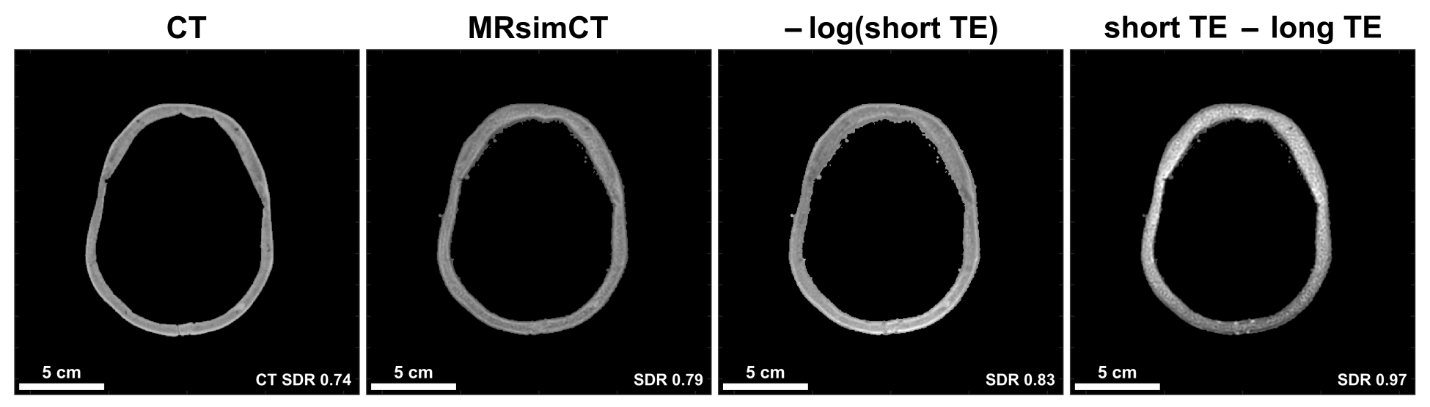


**Supplementary Figure 2.** CT contrast of the skull compared to various MR contrasts (skull C). The units for MRsimCT, – log(short TE), and short TE – long TE are not the same, thus different windowing and leveling were used. Scale bars and skull density ratios (SDRs) are shown at the bottom of each image. Corresponds with Figure 1 in the main text.


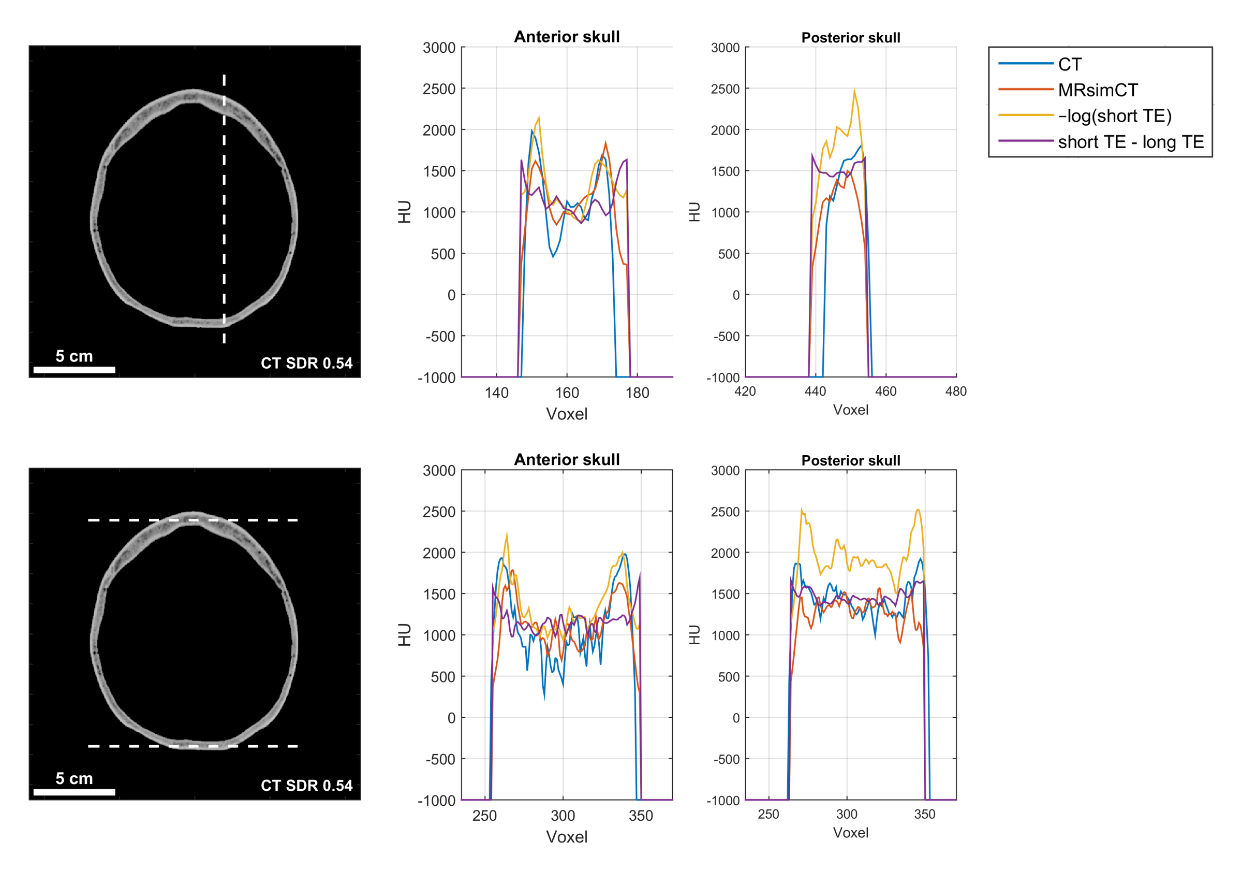


**Supplementary Figure 3.** Cross sections of skull A showing voxel values for the CT and MR contrasts.

**Skull A**


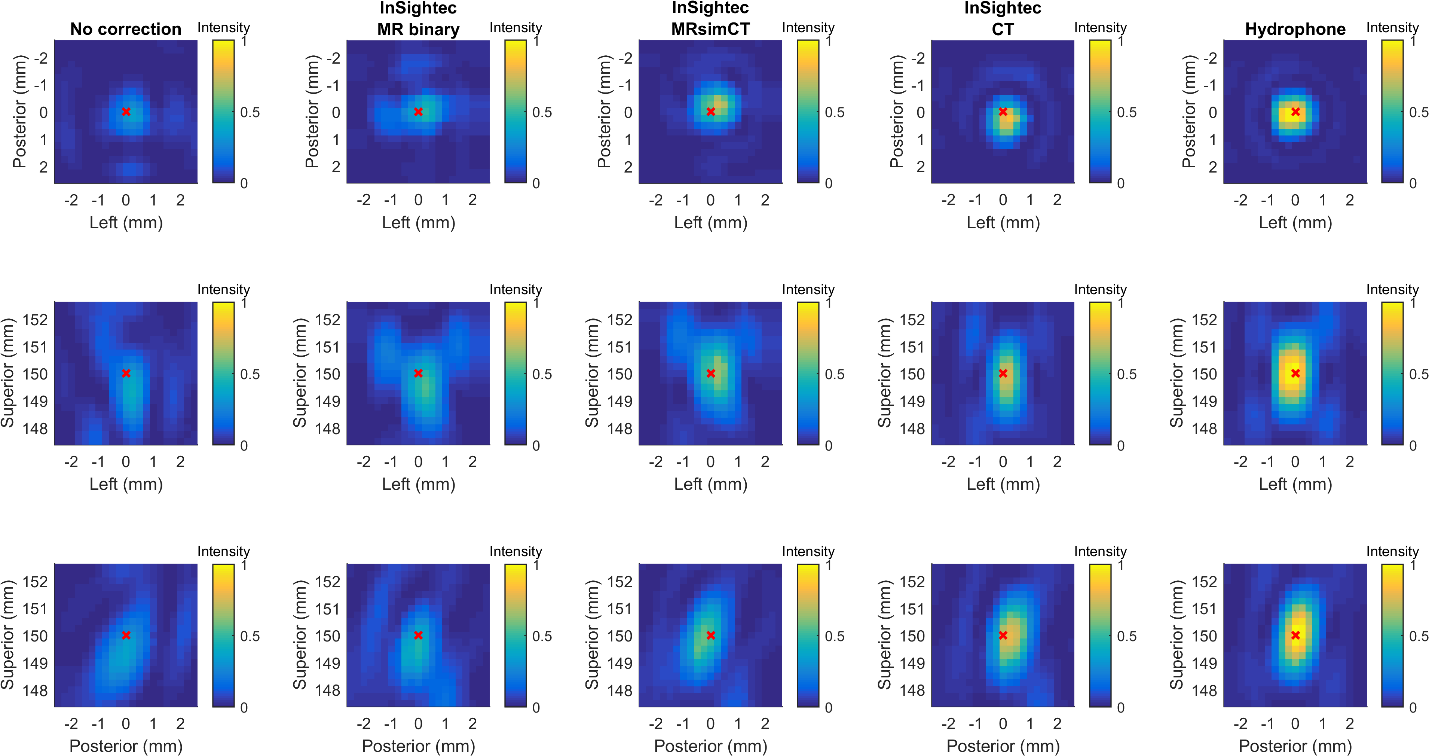


**Supplementary Figure 4.** Three-plane cross sections of the focal spots generated with each phase correction method, while target is at the geometric focus (Skull A). Corresponds with Figure 4 in the main text. The red x marks the location of the targeted position.

**Skull B**


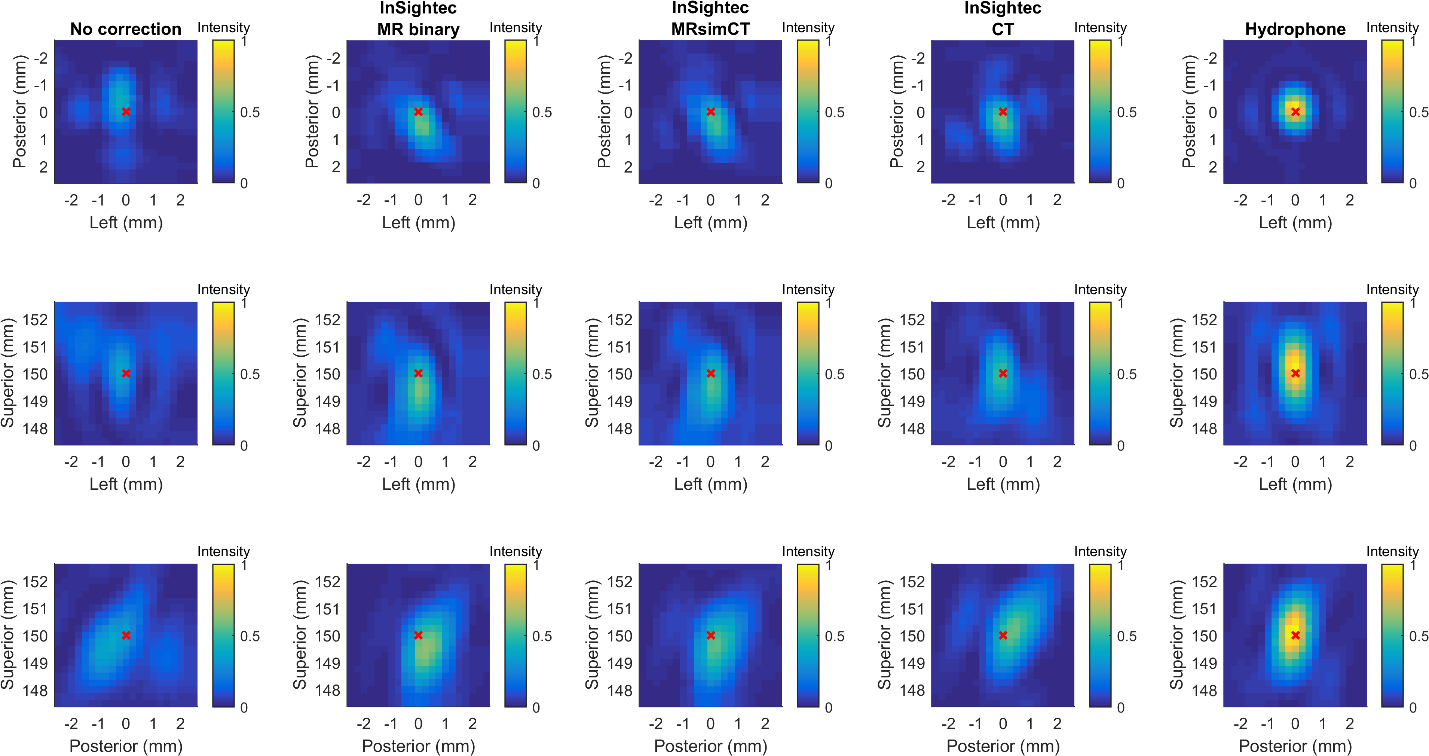


**Supplementary Figure 5.** Three-plane cross sections of the focal spots generated with each phase correction method, while target is at the geometric focus (Skull B). Corresponds with Figure 4 in the main text. The red x marks the location of the targeted position.

**Skull C**


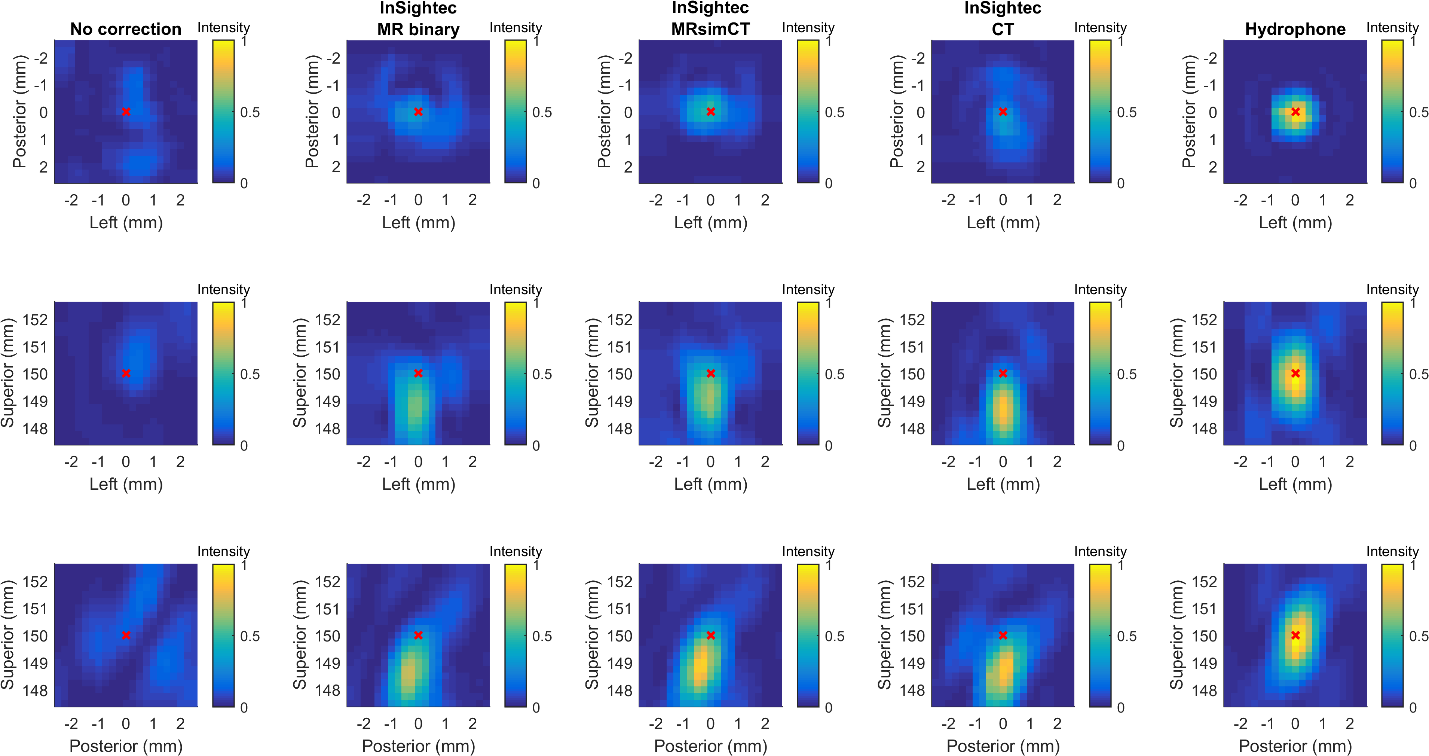


**Supplementary Figure 6.** Three-plane cross sections of the focal spots generated with each phase correction method, while target is at the geometric focus (Skull C). Corresponds with Figure 4 in the main text. The red x marks the location of the targeted position.


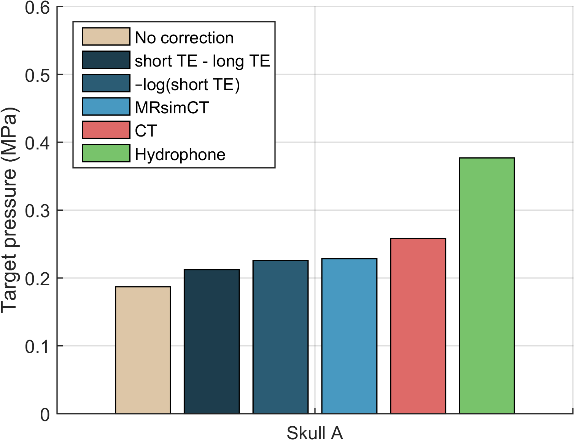


**Supplementary Figure 7.** Phase correction using the different MR contrasts on the single element time series data.

| **Target pressure** | MR binary | MRsimCT | CT |
| --- | --- | --- | --- |
| MR binary | - | 0.573 | 0.879 |
| MRsimCT | - | - | 0.999 |
| CT | - | - | - |

| **Peak pressure** | MR binary | MRsimCT | CT |
| --- | --- | --- | --- |
| MR binary | - | 0.812 | 0.776 |
| MRsimCT | - | - | 0.883 |
| CT | - | - | - |

| **Target intensity** | MR binary | MRsimCT | CT |
| --- | --- | --- | --- |
| MR binary | - | 0.573 | 0.879 |
| MRsimCT | - | - | 0.999 |
| CT | - | - | - |

| **Peak intensity** | MR binary | MRsimCT | CT |
| --- | --- | --- | --- |
| MR binary | - | 0.807 | 0.757 |
| MRsimCT | - | - | 0.907 |
| CT | - | - | - |

| **Distance** | MR binary | MRsimCT | CT |
| --- | --- | --- | --- |
| MR binary | - | 0.269 | 0.768 |
| MRsimCT | - | - | 0.656 |
| CT | - | - | - |

| **Volume** | MR binary | MRsimCT | CT |
| --- | --- | --- | --- |
| MR binary | - | 0.926 | 0.689 |
| MRsimCT | - | - | 0.209 |
| CT | - | - | - |

| **Dice coefficient** | MR binary | MRsimCT | CT |
| --- | --- | --- | --- |
| MR binary | - | 0.344 | 0.740 |
| MRsimCT | - | - | 0.998 |
| CT | - | - | - |

**Supplementary Table 1.** Adjusted p values from Tukey’s multiple comparisons test, which were performed after a one-way repeated measures ANOVA test (target at the geometric focus). No statistical difference was observed between the MR binary, MRsimCT, and CT image inputs.

|  | No correction | InSightec  MR binary | InSightec  MRsimCT | InSightec  CT | Hydrophone |
| --- | --- | --- | --- | --- | --- |
| Target pressure | 0.11 ± 0.04 MPa | 0.17 ± 0.04 MPa | 0.22 ± 0.05 MPa | 0.22 ± 0.08 MPa | 0.31 ± 0.04 MPa |
| Peak pressure | 0.17 ± 0.04 MPa | 0.23 ± 0.02 MPa | 0.25 ± 0.05 MPa | 0.26 ± 0.05 MPa | 0.31 ± 0.04 MPa |
| Target pressure  (normalized to hydrophone) | 36 ± 12% | 54 ± 11% | 69 ± 8% | 69 ± 17% | 100 ± 0% |
| Peak pressure  (normalized to hydrophone) | 55 ± 6% | 74 ± 8% | 82 ± 11% | 84 ± 9% | 100 ± 0% |
| Target intensity  (normalized to hydrophone) | 14 ± 8% | 30 ± 11% | 48 ± 11% | 49 ± 25% | 100 ± 0% |
| Peak intensity  (normalized to hydrophone) | 31 ± 7% | 55 ± 11% | 67 ± 18% | 71 ± 15% | 100 ± 0% |
| Positioning error | 1.21 ± 0.64 mm | 0.91 ± 0.37 mm | 0.59 ± 0.22 mm | 0.61 ± 0.54 mm | 0.00 ± 0.00 mm |
| Focal spot volume | 12.16 ± 9.68 mm^3^ | 6.98 ± 0.73 mm^3^ | 5.70 ± 2.43 mm^3^ | 5.72 ± 2.35 mm^3^ | 3.86 ± 0.24 mm^3^ |

**Supplementary Table 2.** Summary of results for the phase corrected focal spots (target 5 mm left of the geometric focus).


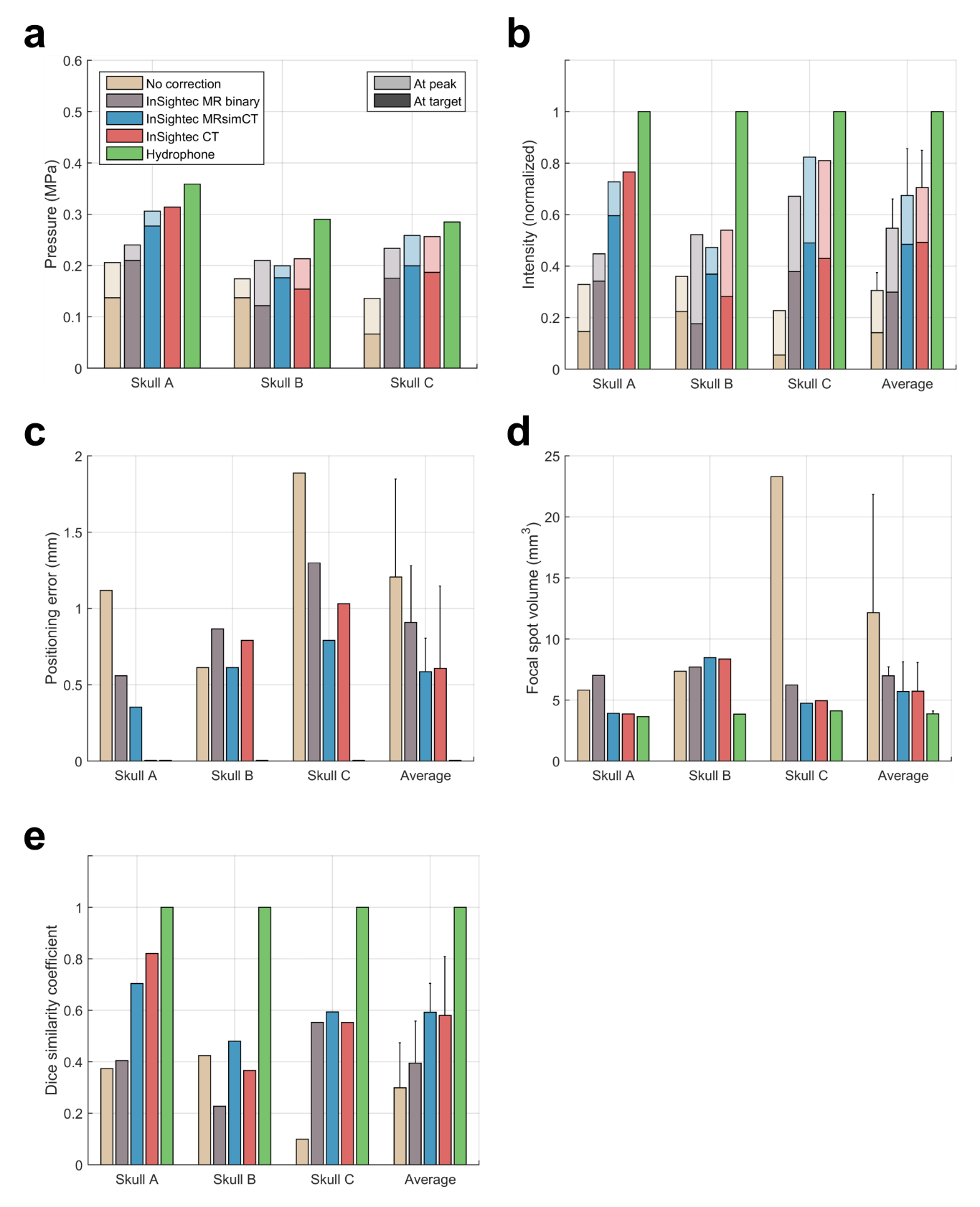


**Supplementary Figure 8.** Beamforming performance of different image types with the target placed 5mm left of the geometric focus. a) Target and peak pressure (shown with darker and lighter colors, respectively) when operating the transducer at 20 W of electrical power. b) Target and peak intensity normalized to the hydrophone method. c) Focal spot positioning error. d) Focal spot volume. Standard deviation bars are shown.

| **Target pressure** | MR binary | MRsimCT | CT |
| --- | --- | --- | --- |
| MR binary | - | 0.184 | 0.554 |
| MRsimCT | - | - | 0.999 |
| CT | - | - | - |

| **Peak pressure** | MR binary | MRsimCT | CT |
| --- | --- | --- | --- |
| MR binary | - | 0.754 | 0.609 |
| MRsimCT | - | - | 0.679 |
| CT | - | - | - |

| **Target intensity** | MR binary | MRsimCT | CT |
| --- | --- | --- | --- |
| MR binary | - | 0.138 | 0.584 |
| MRsimCT | - | - | 0.999 |
| CT | - | - | - |

| **Peak intensity** | MR binary | MRsimCT | CT |
| --- | --- | --- | --- |
| MR binary | - | 0.712 | 0.535 |
| MRsimCT | - | - | 0.721 |
| CT | - | - | - |

| **Distance** | MR binary | MRsimCT | CT |
| --- | --- | --- | --- |
| MR binary | - | 0.217 | 0.437 |
| MRsimCT | - | - | 0.999 |
| CT | - | - | - |

| **Volume** | MR binary | MRsimCT | CT |
| --- | --- | --- | --- |
| MR binary | - | 0.786 | 0.782 |
| MRsimCT | - | - | 0.999 |
| CT | - | - | - |

| **Dice coefficient** | MR binary | MRsimCT | CT |
| --- | --- | --- | --- |
| MR binary | - | 0.358 | 0.639 |
| MRsimCT | - | - | 0.999 |
| CT | - | - | - |

**Supplementary Table 3.** Adjusted p values from Tukey’s multiple comparisons test, which were performed after a one-way repeated measures ANOVA test (target 5 mm left of the geometric focus). No statistical difference was observed between the MR binary, MRsimCT, and CT image inputs.


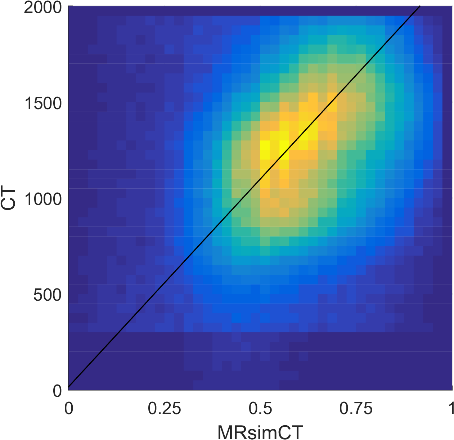

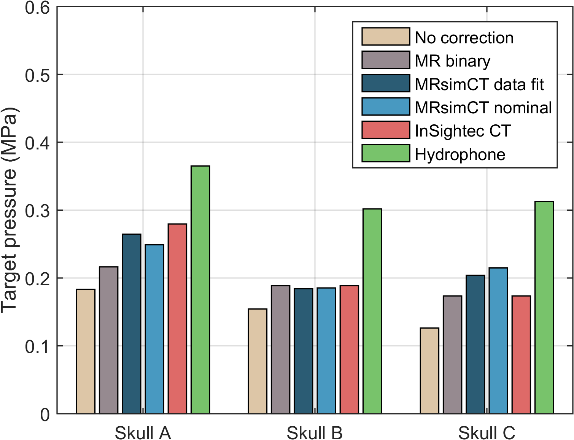


**Supplementary Figure 9.** Phase correction using a linear relationship fit to the data. Results were computed using the post hoc analyses described in the methods, therefore only target pressure was available. There was no systematic error or statistical difference between the linear fit and the nominal linear relationship (dark and light blue, respectively).

**Calculation of HU_bone_**

To justify our use of HU_bone_ = 2000, we show our method for calculating HU_bone_. We performed the calculations using National Institute of Standards and Technology (NIST) reported bone density (ρ_bone_) and mass attenuation coefficients for bone and water ^[47]^. We used the standard Hounsfield unit equation:

${HU}_{bone}= 1000* \frac{{\frac{\mu}{\rho}}_{bone}\rho_{bone} - {\frac{\mu}{\rho}}_{water}\rho_{water}}{{\frac{\mu}{\rho}}_{water}\rho_{water} - {\frac{\mu}{\rho}}_{air}\rho_{air}}$ (Equation 1)

where $\frac{\mu}{\rho}$ is the mass attenuation coefficient and ρ is the density. The mass attenuation coefficients of bone and water are functions of photon energy, therefore HU_bone_ is affected by the CT tube voltage. The material densities were ρ_bone_ = 1920 kg/m^3^ and ρ_water_ = 1000 kg/m^3^.

HU_bone_ as a function of effective tube voltage is shown in Supplementary Figure 8. We assumed an effective tube voltage of 60 kV for a 120 kVp spectrum because the spectrum data was not available to us. The resulting HU_bone_ was calculated to be 1965 and rounded up to 2000.


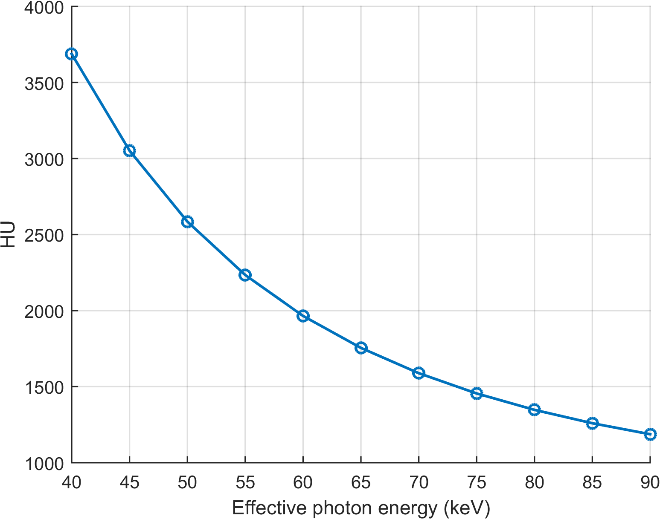


**Supplementary Figure 10.** Calculation of HU_bone_ as a function of effective photon energy. ρ_bone_ and mass attenuation coefficients for bone and water were referenced from the National Institute of Standards and Technology ^[47]^.


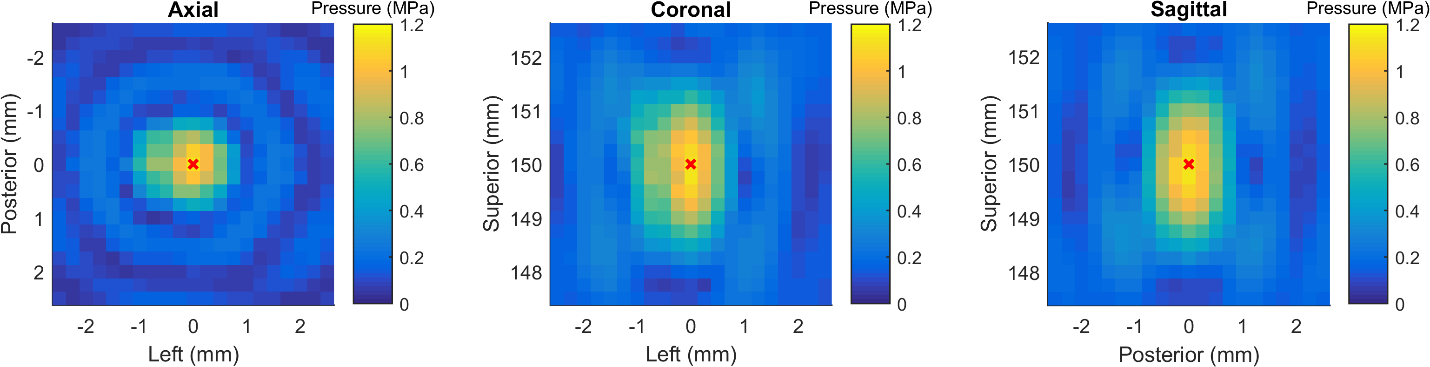


**Supplementary Figure 11.** Three-plane cross section of the focal spot in water using 20 W of electrical power. The red x marks the location of the geometric focus, which was earlier determined using a series of 2D scans to localize the focal spot. In this sonication mode, the InSightec transducer automatically applies phase corrections to account for element positioning.


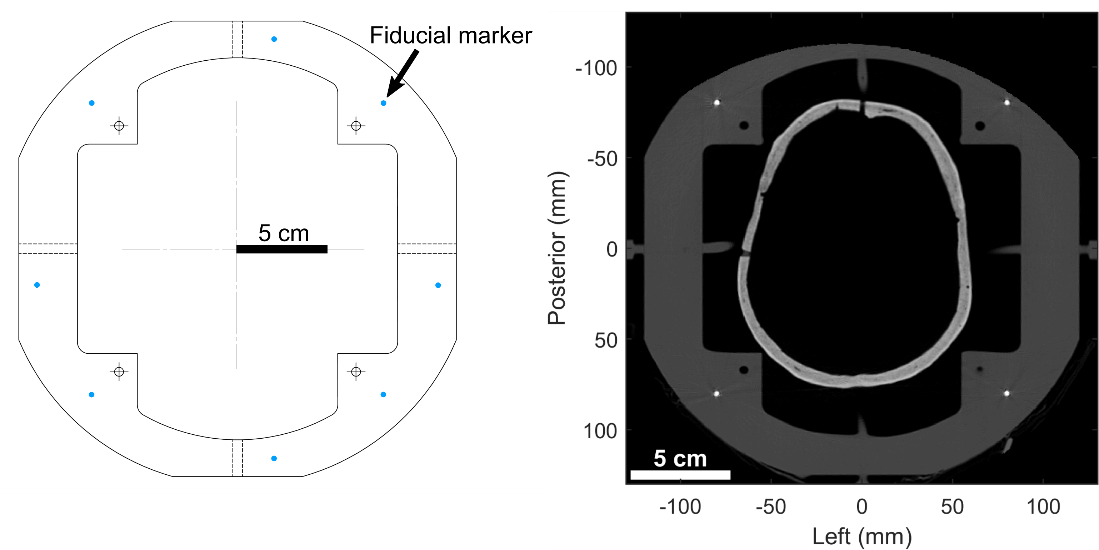


**Supplementary Figure 12.** Registration of CT coordinate space to transducer coordinate space. A singular value decomposition-based least squares registration ^[15,38,48]^ was performed to achieve point-wise registration between CT and transducer positions (Equation 1). Tantalum bead fiducial markers were used for registration. The relative positioning between head frame and transducer are specified in the computer aided design (CAD) file. After performing this registration step, the skull, head frame, and transducer are registered. On the right plot, only four tantalum beads can be seen because the other four are in a different plane.


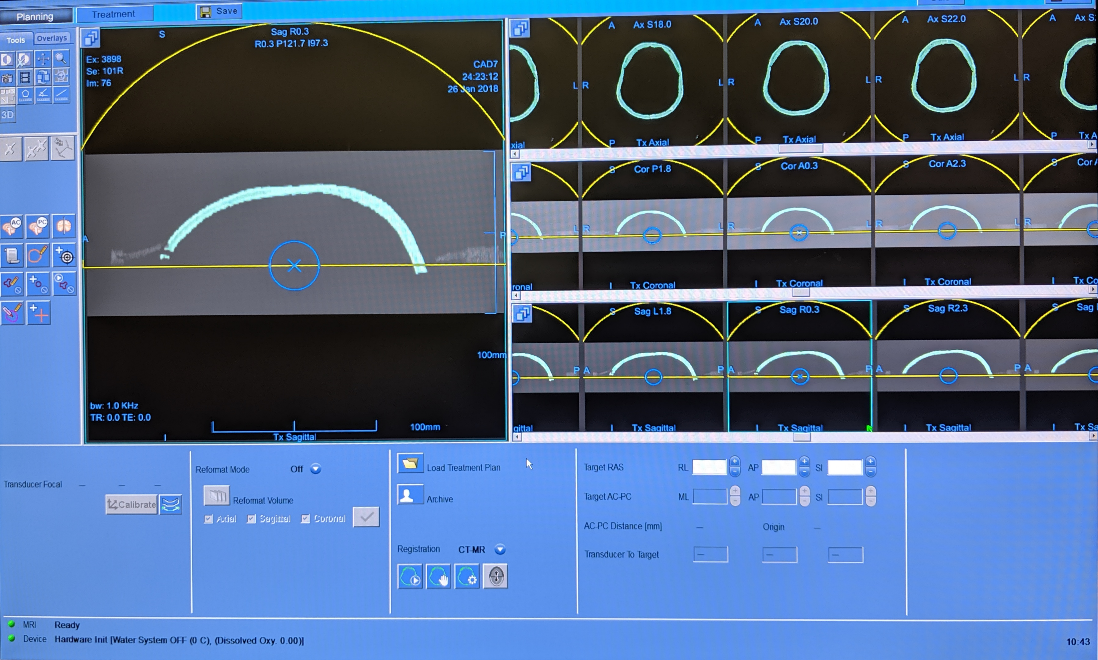


**Supplementary Figure 13.** Screenshot of the InSightec workstation operating in clinical mode. The position of the transducer (yellow outline) and target (blue x) are shown relative to the registered skull. For illustration, the image window and level have been adjusted to show the head frame. When calculating phase corrections, a different set of images with the head frame segmented away is used.
